# Comparison of the Feasibility, Efficiency, and Safety of Genome Editing Technologies

**DOI:** 10.3390/ijms221910355

**Published:** 2021-09-26

**Authors:** Nicolás González Castro, Jan Bjelic, Gunya Malhotra, Cong Huang, Salman Hasan Alsaffar

**Affiliations:** 1 School of Biosciences, Faculty of Science, University of Melbourne, Parkville 3052, Australia; ngonzalezcas@student.unimelb.edu.au (N.G.C.); gunya.malhotra@hdr.qut.edu.au (G.M.); chhu@student.unimelb.edu.au (C.H.); salsaffar@student.unimelb.edu.au (S.H.A.); 2 Biotechnology Department, Environment and Life Sciences Research Center, Kuwait Institute for Scientific Research, Shuwaikh 13109, Kuwait

**Keywords:** meganucleases, zinc finger nucleases, TALENs, CRISPR-Cas, genome editing

## Abstract

Recent advances in programmable nucleases including meganucleases (MNs), zinc finger nucleases (ZFNs), transcription activator-like effector nucleases (TALENs), and clustered regularly interspaced short palindromic repeats-Cas (CRISPR-Cas) have propelled genome editing from explorative research to clinical and industrial settings. Each technology, however, features distinct modes of action that unevenly impact their applicability across the entire genome and are often tested under significantly different conditions. While CRISPR-Cas is currently leading the field due to its versatility, quick adoption, and high degree of support, it is not without limitations. Currently, no technology can be regarded as ideal or even applicable to every case as the context dictates the best approach for genetic modification within a target organism. In this review, we implement a four-pillar framework (context, feasibility, efficiency, and safety) to assess the main genome editing platforms, as a basis for rational decision-making by an expanding base of users, regulators, and consumers. Beyond carefully considering their specific use case with the assessment framework proposed here, we urge stakeholders interested in genome editing to independently validate the parameters of their chosen platform prior to commitment. Furthermore, safety across all applications, particularly in clinical settings, is a paramount consideration and comprehensive off-target detection strategies should be incorporated within workflows to address this. Often neglected aspects such as immunogenicity and the inadvertent selection of mutants deficient for DNA repair pathways must also be considered.

## 1. Introduction 

The previous decade has seen a dramatic surge in the adoption and use of genome editing technologies, from academic research to industrial and clinical applications. This interest has been driven by the development of increasingly versatile and easy-to-use technologies, along with more robust detection methodologies to survey editing activity, both on- and off-target. 

This cycle of continuous innovation has resulted in an increasing number of options and specific variants within each genome editing technology, as well as in significant volumes of information. At the same time, the audience interacting with the field of genome editing has grown and will continue to as more applications begin interfacing with society (e.g., gene drives, agrigenomics, gene therapies). A standard and equitable framework for assessment is, therefore, necessary for understanding the rapidly evolving field and making informed decisions, particularly by stakeholders with distinctly different backgrounds.

In this review, we examine the four main genome editing technologies (meganucleases, zinc finger nucleases, TALE nucleases, and CRISPR-Cas) based on four pillars: context, feasibility, efficiency, and safety. The context for each technology is provided via a brief overview of the biological origins of their underlying components, their overall structure, and the mechanisms enabling their function. Feasibility explores the technical processes necessary to use each technology for a new target, focusing on the need for protein engineering and potential issues that are worth considering. Efficiency presents an overview of the modification rates reported for each technology in the literature, with information regarding factors that can impact it such as the target gene, cell type, and delivery system used. Finally, safety focuses on the previously reported off-target modification rates and existing experimental comparisons. This section also discusses potential causes underlying off-target activity and highlights additional concerns potentially impacting safety in sensitive applications. 

### Double-Strand Breaks and Repair Mechanisms

Genome editing platforms described in this review rely on the core mechanism of introducing a double-strand break (DSB) to the target DNA. Being deleterious to cells unless corrected, DSBs elicit nonhomologous end joining (NHEJ) and homology-directed repair (HDR) responses to mend the breaks [1,2]. It is these repair mechanisms that underlie the ability to modify or “edit” the desired sequence.

While the specific mechanism used depends on several factors, such as the nature of the DSB, cell cycle stage, and chromatin status, NHEJ constitutes a more frequent response and occurs quicker than HDR [3]. During NHEJ, DNA ends are recognised, processed (with or without resection), and joined without a homologous genetic template; in contrast to HDR, which requires a homologous sequence to guide repair [1,3]. These diverging mechanisms result in different efficiency and precision: NHEJ frequently introduces sequence errors in the form of nucleotide insertions and deletions (indels) [4], whereas HDR uses a donor sequence with high similarity and overlap to the original sequence to produce precise, but low-efficiency repairs [2]. 

## 2. Meganucleases

### 2.1. Origin, Structure, and Function

Meganucleases (MNs) are enzymes with long target recognition sequences and high DNA cleavage specificity [5]. They are categorised into two types: homing endonucleases (HEs) and synthetic meganucleases. HEs occur in nature across the mitochondrial and chloroplast genomes of eukaryotes (particularly plants, algae, fungi, and protozoans) and in the genomes of archaea, bacteria, and bacteriophages [6,7]. Meanwhile, synthetic meganucleases are produced by swapping or modifying domains from different HEs [8,9]. 

HEs are mobile genetic elements that naturally propagate by focusing (homing) on target DNAs [7] via long recognition sequences of 14–40 base pairs (bps) [10]. They induce site-specific DSBs and stimulate endogenous repair mechanism pathways of homologous recombination [11]. They are commonly divided into six different families according to variations in structure and sequence motifs: LAGLIDADG, HNH, EDxHD, GIY-YIG, PD(D/E)XK, and His-Cys box [12], all of which are found within well-defined host ranges [13]. 

**Figure 1:**
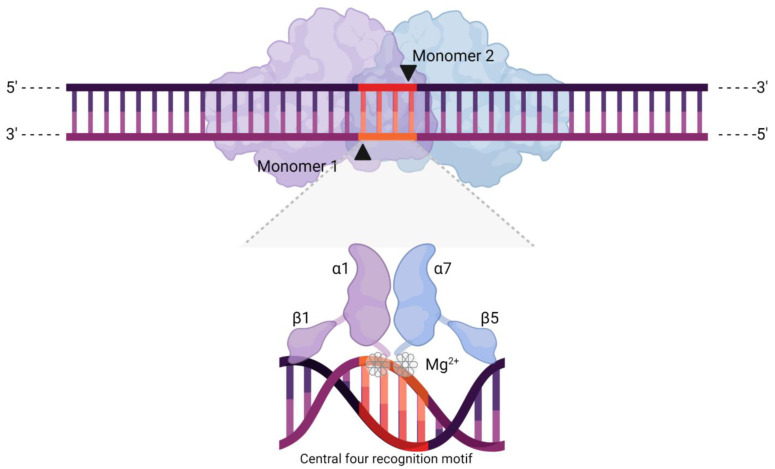
Schematic representation of a meganuclease (MN), depicting its two characteristic ɑββɑββɑ domains, shown here as monomers of different colour. The arrows indicate the cleavage sites, resulting in a DSB with 4-nt 3′ overhangs. The highlighted section represents the central four recognition motif, shown at the bottom in close proximity to the MN active site. This set of four bases lies at the centre of the broader DNA recognition sequence and directly determines cleaving activity and specificity through a cofactor displacement mechanism. Effectively, mismatches in this motif produce structural rearrangements that displace metallic ions (Mg^2+^) from the active sites.

Structurally, all meganucleases comprise two ɑββɑββɑ domains, either simultaneously present in a multidomain protein or brought together via a homodimerisation domain [7] (Figure 1). In nature, MN activity essentially consists of recognising and then splicing a target region [14], which has been well-studied in the LAGLIDADG family of MNs. In fact, members of this family feature distinct functions, such as endonucleases that recognise and cleave exons [12]. 

Initially, LAGLIDADG MNs interact with their targets via nonspecific contacts between β strands and the backbone of the target DNA. As the β strands are physically inserted into the major groove, clusters of 6 to 9 amino acids termed “contact modules” recognise 2–4 bps of DNA [5,6]. At this point, it is the structural and spatial features of the target DNA region that determine the high specificity of MNs in a cofactor displacement-based process known as the “indirect readout mechanism” [5,15]. Essentially, upon binding their target, meganucleases bend the DNA to bring the central four recognition motif in close proximity to their catalytic site, with the energetic cost of such action changing as a function of the base pairs present [5,12,15,16]. This recognition motif is a set of four bases (usually two A–T or T–A pairs in wild-type MNs) at the centre of the target sequence [5], and which ultimately determines the cleavage specificity of an MN, with mismatches precluding activity [17]. The indirect readout mechanism underlies such tight regulation and involves a series of small changes in base pair positioning and interaction with the meganucleases’ residues, generating a knock-on effect in response to a mismatch. Besides altering the energetic cost of unstacking bases (penalties), positional changes caused by a mismatch effectively displace a metallic ion cofactor (usually Mg^2+^) from its regular location at the active site, preventing cleavage [5,15]. However, when there are no mismatches, the meganuclease will bind to DNA, bend it, and introduce a cleavage across its minor groove at the central four motif, producing 4-nt 3′-OH overhangs [12].

### 2.2. Feasibility

Developing a new MN variant that specifically targets a new sequence (retargeting) requires a significant amount of protein engineering, mutant screening and selection, and optimisation procedures [6,15,16]. This is due to the dependence of MNs on the presence of a central motif, their combination of binding and cleaving functions within the same module, and the indirect readout mechanism involving nonspecific contacts between the protein and the DNA backbone [5,16]. Therefore, any specific mutations aimed at improving sequence specificity could influence cleaving efficiency and vice versa. 

**Figure 2:**
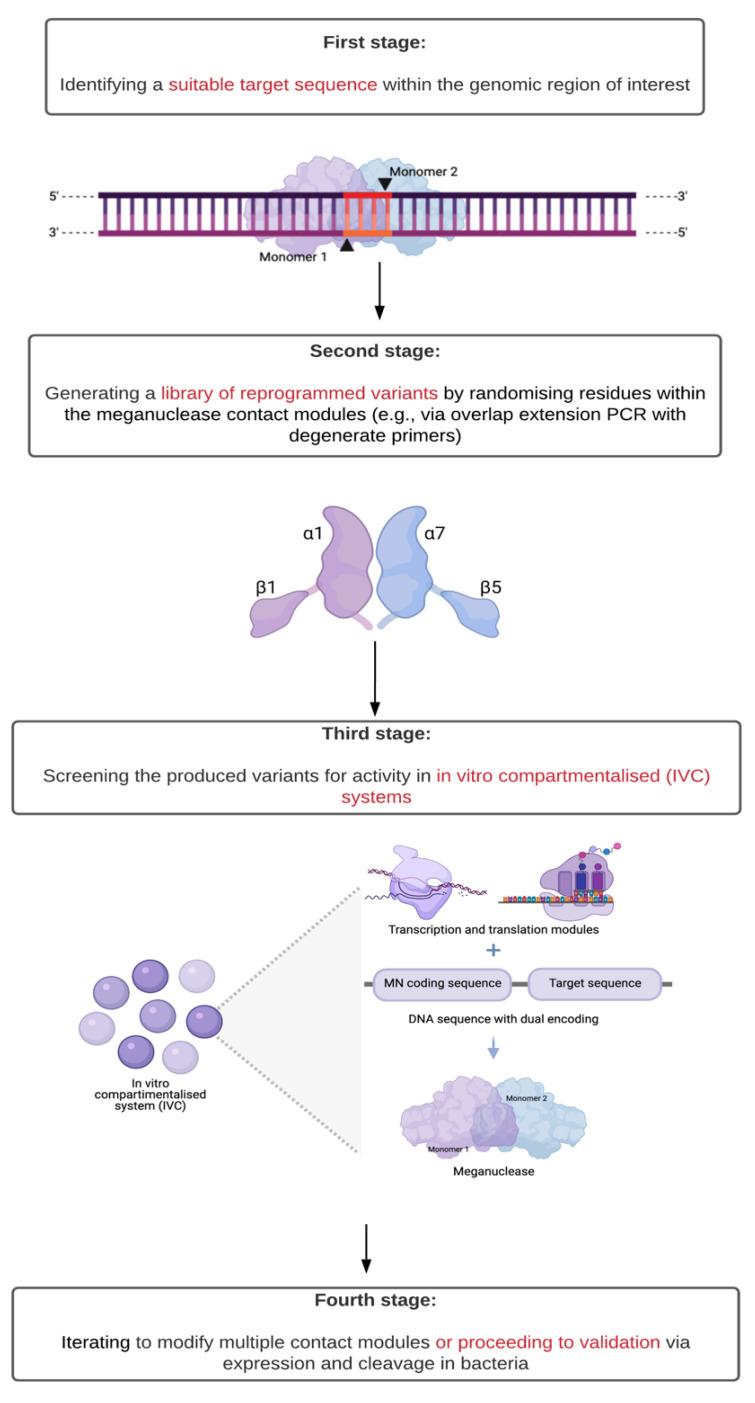
Workflow for reprogramming the specificity of a meganuclease (MN) [6,18]. In the first stage, the target sequence must meet two conditions: (1) contain a central four recognition motif that matches the meganuclease to be modified and (2) have a minimal number of mismatches as compared to its original recognition sequence. In the second stage, this selection minimises the number of contact modules to be modified (represented here with the distinctive structure of the MN active sites). The coupled transcription and translation capabilities of in vitro compartmentalised systems (IVC) along with sequences encoding both the meganuclease variant and its target enable high-throughput screening of activity under increasingly stringent conditions.

In general, protein engineering for reprogramming an MN occurs over four stages (Figure 2). First, a suitable target sequence within the genomic region of interest is identified as both containing a central four recognition motif that matches an existing wild-type or previously engineered MN and having the least number of mismatches as compared to the original recognition sequence. Second, an extensive library of reprogrammed variants is generated by randomising residues in the contact modules of the selected meganuclease. Third, the produced variants are screened for activity in in vitro compartmentalised (IVC) systems: independent aqueous droplets with coupled transcription and translation capabilities and which carry DNA encoding both the MN and its target sequence. Multiple rounds of screening and selection are then performed, with increasingly stringent criteria: time for synthesis, time for cleavage, and temperature resistance [6,18]. Finally, this process can proceed iteratively to modify multiple contact modules or move on to validation of activity via expression and cleavage in bacteria.

An alternative approach to retargeting is the MegaTAL (MT) concept arising from the fusion of a transcription activator-like effector (TALE) domain to a meganuclease. MegaTALs have high specificity, increased modification rates, and decreased off-target activity due to the presence of two recognition modules [17,19,20]. This approach offers the advantage of reducing the burden of extensive protein engineering of an MN for a new sequence. Moreover, it adds multiple modular components whose variation can help fine-tune activity.

Finally, it is worth noting that the distinctive features of MNs make them a useful tool for certain genome editing applications. For instance, their small size (180–440 residues, 18–40 kDa, coded in sequences of ~1 kb), nonrepetitive sequence, and monomeric nature, make them amenable to packaging and delivery as plasmids, mRNA, viral vectors, and proteins [7,13,19,21]. Meanwhile, the high cleaving specificity from long target sequences (12–40 bp) and the indirect readout mechanism can be considered an advantage for highly specific applications, especially when the selected MN is part of a MegaTAL architecture [19,20,22]. 

### 2.3. Efficiency 

The on-target efficiency of MNs is highly variable and dependent upon the MN selected, the cell type, target sequence, and use, whether in vivo or ex vivo (Table 1). For instance, the ex vivo modification rate of *COL7A1* in a recessive dystrophic epidermolysis bullosa (RDEB) cell line of human primary fibroblasts when using a specific I-CreI MN variant was 2.2% [21], while the modification rate was up to 6% for the *RAG1* gene in the 293 human cell line [23].

Similarly, efficiency rates can change significantly when MNs are used as part of novel architectures, such as MegaTALs. Case in point, modification rates have been shown to change from as little as 1.6% for a standalone MN targeting T cell receptor alpha (*TCRα*) to as high as 70.4% when a MegaTAL is used to target the same gene [19].

In general, however, the modification rate of purpose-modified solo MN variants is usually below 10% [19,21,23,24]. 

### 2.4. Safety

Cytotoxicity associated with MNs has been characterised as insignificant in several applications [21,23,27] and, despite being present, off-target activity has been reported to be low [19,20,24]. However, the source species of the meganuclease used, the cell type of interest, and the method of delivery influence specificity and off-target activity [21]. Moreover, as is the case for all genome editing platforms, MNs retain the potential to introduce mutations ranging from indels to gross chromosomal rearrangements, producing cytotoxicity in targeted cells [21,27]. Despite this, the 4-nucleotide 3′ overhang introduced during cleavage particularly promotes HDR [13,17,28] and, coupled with an origin in non-pathogenic organisms, constitutes a significant advantage for MN use in sensitive applications.

Off-target activities of MNs can also be influenced by the type of ion used as cofactor. It has been reported that manganese can substitute magnesium in certain MN variants and render them more tolerant of single substitutions in the target, potentially rescuing cleaving activity in a mismatched sequence [5]. Therefore, the presence of manganese should be considered in applications that require high specificity.

MNs are also intrinsically tolerant of substitutions in their target sequence at the binding level [6,16,17]. Beyond off-target cleaving (which is limited by the indirect readout mechanism), this can impact editing efficiency (modification rates) because MNs may imprecisely bind highly similar targets at different positions in the genome, disabling them from performing on-target activities [6].

Finally, despite significantly increasing efficiency, the MegaTAL architecture itself has pitfalls. TALE addressing can worsen off-target activity for those sites that are already susceptible to MN activity, and which are not normally affected because of low affinity [19]. However, MegaTALs will not increase off-target effects for sequences that are not already susceptible to MN activity.

## 3. Zinc Finger Nucleases

### 3.1. Origin, Structure, and Function

**Figure 3:**
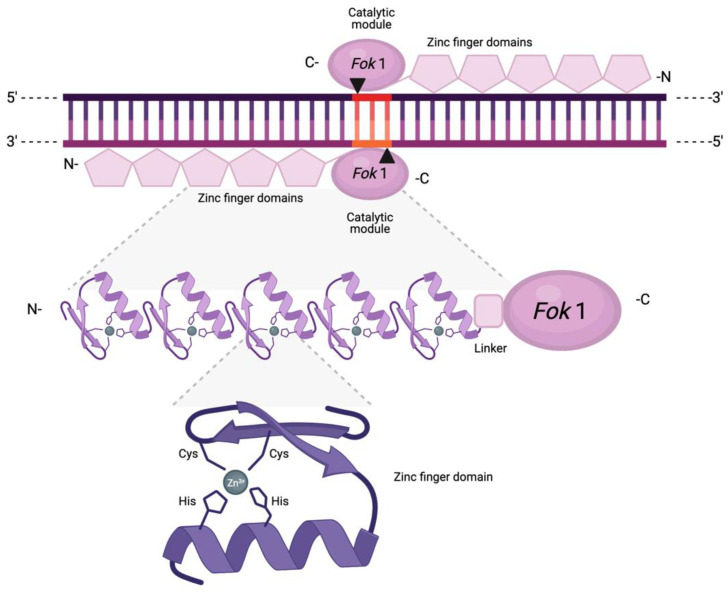
Schematic representation of a zinc finger nuclease pair with distinct binding (ZF motifs) and cleaving (FokI) domains. Each ZF motif comprises a conserved set of two cysteines (Cys) and two histidines (His), which interact directly with a central zinc ion. This interaction, along with the dual beta sheets and single alpha helix, result in the characteristic protruding structure. The arrows and the highlighted region represent the resulting DSB and 3-nt 5′ overhangs.

Zinc finger nucleases (ZFNs) are artificial and customisable nucleases arising from the fusion of two functionally distinct domains. At the N-terminus lie 3–6 zinc finger motifs (ZFs). Originally identified in the transcription factor IIIA present in *Xenopus* oocytes, these motifs are engineered to enable specific DNA recognition and binding [29,30,31]. This ZF domain is fused at its C-terminus via a peptide linker with the nonspecific type II restriction enzyme FokI derived from *Flavobacterium okeanokoites* (Figure 3) [32]. This domain is responsible for DNA cleavage.

Each ZF motif contains approximately 30 amino acid residues and forms a ββα structure, with a crucial Cys_2_/His_2_ (C2H2) repetition directly interacting with a central zinc ion that stabilises and coordinates the protruding finger-like structure [31]. The sequence specificity of ZFs arises from the capacity of each motif to recognise distinct segments of approximately 3 bps in the major groove of the DNA, which enables ZFN customisation as novel sequences can be targeted through the identification of motifs with an affinity for a defined triplet [32,33]. In fact, there have been several attempts at constructing modular assembly libraries to target all 64 possible nucleotide triplets [34,35]. However, while in principle ZFNs can be designed to bind and cleave arbitrarily chosen sequences, generating ZFs with high specificity has shown limited success. The production and selection of active ZFNs is dependent on significant optimisation, and the modification of sequence specificity is often laborious and time-intensive.

Depending on the number of ZF motifs, a single ZFN could recognise a specific target site of 9–18 bps [33]. However, given the obligatory dimerisation requirement of FokI, two ZFNs must bind simultaneously in opposite orientations for a DSB to occur [36]. FokI can then introduce a DSB featuring 3-nucleotide 5’ overhangs. This contrasts with the 4-nt 3′ overhangs from MNs, blunt ends from Cas9, and 5-nt 5′ overhangs from Cas12a (Cpf1) [17].

### 3.2. Feasibility

The development of a new ZFN variant to target a particular DNA sequence is generally regarded as laborious, time-consuming, and contingent on expertise in protein engineering, context-dependent assembly, and enhancement techniques [7,31]. This is due to the absence of a consistent ZF-base “code” and the variation in sequence recognition caused by interactions with neighbouring ZFs [7,37]. As a result, ZFN engineering focuses on selecting compatible ZF combinations that display adequate efficiency and specificity together [38]. Some of the main approaches are summarised in Table 2.

Moreover, the number of actual sites that can readily be targeted by ZFNs is limited. There is a low number of loci that can be successfully targeted by ZFNs in mammalian cells [31,39], and potential target sites are estimated to occur at a frequency of 1 per 500 bp [40]. Furthermore, ZFNs exhibit a preference towards GC-rich sequences [17,29,41].

Despite these challenges, ZFNs feature desirable traits that enhance their ease of use once they have been developed, optimised, and validated for a specific target. Their small size (~40 kDa) makes them compatible with many delivery methods, from plasmids to adeno-associated viral (AAV) vectors [7]. They do not require additional components for targeting or cleaving and do not experience assembly issues during packaging, as is the case for TALENs in lentiviral vectors (LVs) [44,45]. ZFNs also enable the use of longer recognition sequences (proportional to the number of ZFs used) and do not harbour immunogenic epitopes despite the bacterial origin of FokI [46]. Moreover, the short peptide linker can be modified to fine-tune the genome editing activity, from efficiency to off-target activity [31,44,47], and is the basis of base-skipping protein architectures that allow highly specific base targeting. Finally, FokI itself is compatible with mutations that significantly improve the efficiency and specificity rates to levels adequate for clinical applications [48]. 

### 3.3. Efficiency

The efficiency of a given ZFN pair depends on its binding affinity and sequence specificity, both of which impact long-term stability and on-target modification [49]. Crucially, affinity and specificity are directly affected by the number of engineered zinc finger motifs: optimal activity has been observed for pairs with 3 + 3 and 4 + 4 zinc fingers as opposed to 5 + 5 and 6 + 6 [39]. While the versions with a greater number of ZF motifs would theoretically provide greater specificity by increasing the size of the target sequence recognised, they also decrease efficiency [32]. Interestingly, studies have also revealed that the higher affinity of ZFNs does not directly correlate with higher activity [33]. These observations imply that the activity of ZFNs is not solely related to the number of ZFs but rather to the balance between their affinity and specificity. 

Besides low DNA-binding levels and reduced specificity, low activity of ZFNs could also be explained by increased cell toxicity. Therefore, while more ZF motifs do not translate into efficiency, the higher specificity can lead to reduced toxicity and, consequently, higher modification rates from the surviving modified cells [32,33,37]. Crucially, the modification rates for ZFNs, expressed as disruption or indel frequencies and interpreted as the successful introduction of a DSB in a target sequence, are reported to be between 1% and 20% (Table 3) [50]. 

### 3.4. Safety

Several variables influence the off-target activity of ZFNs. For instance, Cornu et al. (2008) reported an inverse correlation between the DNA-binding specificity and the ZFN-associated toxicity [50]. It has also been reported that modulating spacer length can reduce off-target activity [31], and that excess binding energy contributes to off-target ZFN cleavage [54]. Crucially, the presence of paralogues or pseudogenes flanking target sequences in complex genomes is common, and multiple copies of a sequence highly related to the target sequence can result in off-target activity [38]. However, this is a factor affecting all genome editing platforms.

One major mechanism underlying off-target effects of ZFNs is based on their ability to form both homo- and heterodimers. Monomers of a ZFN pair can bind not only to their 18–36 bp target sequence, but also to palindromic sequences based on one of the monomer-binding half-sites, forming a homodimer at a nontarget site. Likewise, a single monomer could bind to several sites bearing sequence similarity to its intended half-site and then form a heterodimer with another monomer in a solution [37,55]. 

Off-target activity of this genome editing platform also stems from the fact that ZFN recognition of a target site is not only determined by the 3–4 bp of DNA interacting with each ZF motif, but also by the interaction between adjacent fingers [7,55]. This context-dependent form of DNA binding can increase the probability of targeting undesired sites. Additionally, some ZFNs have a high tolerance for mismatches in their target sequence, resulting in binding to off-target sites that share as low as 66% identity with the desired site [40].

While the mechanisms leading to homodimerisation have been mitigated through the development of obligate heterodimers [37,56], the overall specificity of ZFNs remains inconsistent across target sites and specific applications. For example, for the allelic disruption in the *BCL11A* locus, ZFNs have been reported to cause less off-target activity as compared to TALEN and CRISPR-based genome editing [37]. However, a parental ZFN pair without reengineering showed approximately 41% indel mutation frequency at off-target sites when targeting the *AAVS1* safe harbour locus [48]. Meanwhile, a *CCR5*-specific ZFN exhibited over 12% of gene disruption frequency at some off-target sites, with this value being almost as high as the frequency of desirable activity at the *CCR5* locus itself [41].

Despite the drawbacks in off-target activity, ZFNs do possess features that can be used for improvement. Beyond obligate heterodimer FokI variants, the optimisation of the linker sequence can reduce off-target activity by limiting the tolerance of the architecture for a mispositioned FokI dimer (i.e., binding at the right sequence, but cleaving elsewhere due to a tolerant linker) [40]. Likewise, constructing a ZFN with a destabilised asymmetric interface has been shown to be less toxic while maintaining the same performance [56]. This makes ZFN activity more dependent on DNA binding and prevents it from forming dimers in a solution. In addition, the allosteric activation of FokI has also been proposed as a viable strategy for specificity improvement via cleavage regulation [31]. 

It has also been reported that modifications of residues linked to the cleavage kinetics of FokI rather than to sequence recognition or cleavage itself can preserve full on-target activity while significantly reducing off-target activity [48]. Miller et al. (2019) reported a modification rate of >98% of human T cells with no detectable off-target activity when implementing ZFN variants targeting the *TRAC* locus and bearing substitutions that attenuate FokI cleavage kinetics [48]. The underlying mechanism proposed is that slower kinetics reduce off-target activity by making cleavage more compatible with sequence-specific dissociation constants. Effectively, ZFs will dissociate from off-target sequences without FokI introducing a DSB if sufficient time is provided. Notably, this concept is not restricted to ZFNs and could be explored in both TALENs and CRISPR-Cas.

Whilst this level of efficiency and specificity has only been achieved for the *TRAC* locus (with the kinetics of the attenuation process having to be repeated for each new target), the result shows the potential of zinc finger nucleases for highly specific and efficient activity at a level viable for clinical application provided sufficient optimisation and engineering have been undertaken for the target sequence.

## 4. TALENs 

### 4.1. Origin, Structure, and Function

**Figure 4:**
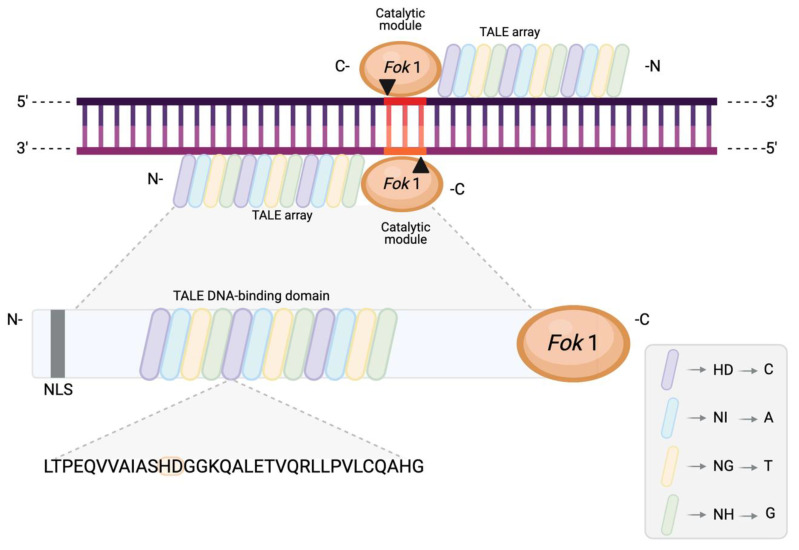
Schematic representation of a TALEN pair with its distinct binding and recognition domain (TALE array) and cleaving domain (FokI). The arrows and the highlighted region represent the resulting DSB and 3-nt 5′ overhangs. Each TALE array contains 15–19 individual tandem repeats, each comprising a conserved sequence of 33–35 amino acids that differ only at amino acid positions 12 and 13. These two amino acid residues are known as the repeat variable diresidue (RVD) and determine the nucleotide binding specificity. The inset presents a small subset of the RVD base “code” for illustrative purposes. NLS, nuclear localisation signal.

Transcription activator-like effector nucleases (TALENs) are artificial restriction enzymes that combine the catalytic module of FokI nucleases with the DNA-binding domain of TALEs (Figure 4). TALEs are naturally occurring virulence proteins secreted by plant pathogenic *Xanthomonas* bacteria [40] which bind to specific DNA sequences via a central DNA-binding domain to activate the expression of a host gene. This central domain identifies a target sequence and uses a distinct type of DNA-binding mechanism based on one-to-one correspondence between an individual repeat and a single base. This is made possible by the composition of the domain, which features 15–19 highly similar tandem repeats assembled as an array, with each repeat comprising 33–35 amino acid motifs. The repeat closest to the C-terminus contains only 20 amino acids and is called a “half”-repeat [57]. 

TALE repeats are highly conserved and differ only at amino acid positions 12 and 13, which are known as the repeat variable diresidue (RVD) [57]. The presence of RVDs is what determines the DNA binding specificity of TALE arrays via one-to-one binding of a single repeat to a single nucleotide in the target sequence, with the base specified by the RVD and a preceding thymine at position 0. Twenty-five different RVD types are naturally known, of which the most common ones are HD, NH, NI, and NG, specific for identifying cytosine (C), guanine (G), adenine (A), and thymine (T), respectively (Figure 4) [57]. 

From a structural point of view, the RVD sits between the two *ɑ*-helices present in each repeat and forms a loop that directly interfaces with DNA. However, repeat variable residue 2 (RVR2) is the only component that interacts with the DNA base and establishes hydrogen bonding with it at the major groove, thus determining the base specificity. Meanwhile, repeat variable residue 1 (RVR1) indirectly supports DNA binding, and influences efficiency and specificity, by stabilising the loop through an interaction with the amino acid in position 8 of the same repeat. Individual repeats also bind the DNA backbone via amino acids 14–17, contributing to DNA affinity [44,57].

### 4.2. Feasibility

The simplified code used by TALENs for sequence recognition is an advantage in terms of targetability and redesign, especially when compared with MNs and ZFNs. Individual repeats with defined RVDs can be put together into a tandem array, allowing the straightforward construction of a custom DNA-binding domain [41,58]. This is further facilitated by a detailed set of rules that guide the design of TALEs [45].

Nonetheless, TALENs possess challenges of their own, starting with design constraints for the TALE arrays. Both the recognition specificity and the binding affinity of individual RVDs vary significantly, with some exhibiting high binding affinity for a given base but simultaneous degeneracy, while others feature single-base specificity but low binding affinity [44,45]. This heterogeneity among RVDs is accompanied by limitations in reliably recognising guanosine. RVDs that target it often display degeneracy in terms of recognising additional bases and, in the case of more specific variants (e.g., NH and NK), reduced activity [45]. Moreover, methylation of cytosine can alter recognition by its normal RVD and potentially abrogate TALE binding altogether (a different RVD capable of recognising 5′-mC would be required) [41,44,45]. Lastly, reminiscent of the central four recognition motif in MNs and the protospacer adjacent motif in CRISPR-Cas discussed later, conventional TALEs require a thymine to be located at the 5′ end immediately adjacent to the targeted sequence [40,44,45]. This is due to a conserved tryptophan in the N-terminus of TALE repeats that interacts with the thymine’s methyl group [44]. Whilst modified TALE scaffolds have eliminated this requirement, it remains a feature to consider during array selection and development, especially as some of the modified versions exhibit reduced DNA-binding activity [45].

The crux of targeting a new sequence using TALENs is in the complex process for assembling the array itself [29,59]. The most common approach relies on the Golden Gate cloning method, where individual plasmids encoding specific RVD-containing repeats are first amplified, isolated, and purified [59]. The repeats are then separated via digestion from their individual plasmids and sequentially ligated in the order and number required for the intended target sequence [20,45,59]. The numerous steps for producing a new array, involving multiple instances of bacterial transformation, plasmid purification, as well as digestion and ligation reactions, make this process time-consuming. Furthermore, the throughput of the Golden Gate method is limited by the maximum number of repeats that can take part in each digestion/ligation reaction [59]. 

Due to the complexity and limitations of conventional Golden Gate cloning, alternative methods have been developed to simplify the assembly process. Solid-phase assembly and ligation-independent cloning have been used and are compatible with high-throughput workflows [45]. Recently, Zhang et al. (2019) developed a novel plasmid-free library and bacteria-free assembly pipeline in order to facilitate the process to a point comparable to CRISPR-Cas [59]. In this system, four circular pentamers are independently produced from linear dsDNA fragments encoding individual repeats via digestion/ligation reactions. A single-expression plasmid bearing the full sequence of the TALE array is then produced in a single Golden Gate reaction involving the four pentamers and a backbone. This pipeline is estimated to reduce the assembly process to 1 day and reduces the number of the necessary bacterial transformation, colony identification, and validation procedures.

There are also additional challenges due to the repetitive nature of TALEN DNA-binding domains. The high sequence similarity between the tandem repeats can lead to instability of the coding region and even unexpected rearrangements in the TALE array, especially when lentivirus is used as the delivery method [44,45]. While this issue can be ameliorated using recoded TALEN constructs, additional optimisation procedures would be required for each new target gene [45]. Moreover, this repetitive nature can result in individual TALENs having long sequences encoding the arrays, with approximately 2.3 kb required for an 18 bp target site. When considering additional control elements that are necessary for expression, the size of a single TALEN can increase to 4.4 kb, which is at the upper limit of the packaging capacity of the commonly used AAV vectors [44]. This means that the simultaneous delivery of the two TALEN monomers necessary for inducing a DSB would require either two separate AAVs or an adenoviral vector [45].

### 4.3. Efficiency

The specificity in binding offered by TALENs is accompanied by a variable efficiency that is affected by the cell type, specific target sites, duration of effect, and delivery system used.

However, there are strategies to increase TALEN modification rates based on structural changes. For instance, truncating TALE scaffolds on both sides of the repeat units may improve protein stability or place the catalytic centre in a more proper position [60]. This strategy resulted in a 20% increase in the modification rate, with a simultaneous reduction in cytotoxicity with respect to ZFNs, as observed in the *CCR5* and *IL2RG* human loci [40]. A separate comparison between TALENs and ZFNs found similar results, with optimised TALEN scaffolds shown to induce allele modification rates of up to 30% across three human loci tested [41]. The efficiency rates from additional studies are described in Table 4. 

The efficiency of TALENs can be further improved by the creation (via multiple rounds of cycling mutagenesis and DNA shuffling) of highly active FokI variants, as well as using fluorescence-activated cell sorting for the enrichment of edited cells [58]. Genome editing efficiency can also be enhanced by improving how TALENs are delivered to cells. Using a bicistronic TALEN construct which transcribes two proteins from the same plasmid, about 15% higher cleavage activity of TALENs was achieved as compared to their separate expression as two monomers in cotransfection [58]. Furthermore, bicistronic TALENs enable real-time monitoring of transfection efficiency and rapid enrichment selection of nuclease-containing cells [61]. 

On the other hand, it is also important to note that some modifications enhancing specificity can constrain efficiency. For instance, reengineered TALE proteins can have a significantly reduced activity when certain RVDs are used (e.g., NH and NK, which are used for the recognition of guanosine) [45].

### 4.4. Safety

Similarly to ZFNs, the off-target activity of TALENs is affected by tolerance to mismatches, binding to highly similar off-target sites, the homodimerisation potential of the unmodified FokI, and variance in specificity of the short linker between the sequence recognition and cleavage domains [40,41,68]. However, compared with ZFNs, TALENs generally show a higher genome editing specificity as they have fewer context-dependent DNA-binding effects [55]. This means that due to the high specificity of RVDs, TALENs can be flexibly constructed to specifically target desirable sequences with less off-target activity and cytotoxicity [69]. 

Moreover, a comparison between ZFNs and TALENs found that the use of TALENs at the *CCR5* and *IL2RG* loci caused significantly less cytotoxicity [29,40]. The two *CCR5*-specific TALEN pairs only had a mutation frequency of 0.12% at the total off-target sites, while this value increased tenfold when using *CCR5*-specific ZFNs [41]. Similarly, in an *AAVS1*-targeting experiment, the TALEN pair performed better than the ZFN pair. The former resulted in only 0.13% of mutation frequency at one off-target site, whereas the latter caused mutation frequency of around 1~4% at several undesirable loci [41].

Apart from improved specificity in comparison with ZFNs, TALENs also perform well in other off-target evaluation studies and in comparisons with CRISPR-Cas. For example, analysis of TALEN-based editing at four different human loci found the off-target cleavage was undetectable via the IDLV assay [55]. Similarly, despite the observation of off-target cleavages by high-throughput genome-wide translocation sequencing (HTGTS), the frequency of these TALEN cleavages was lower than the frequency of off-target events caused by CRISPR-Cas [55]. Other research using whole-genome sequencing techniques for off-target analysis has reported minimal off-target events and cytotoxicity for TALENs [69]. Their editing specificity has even shown greater capability than CRISPR-based editing in reducing the safety risk in some disease treatment applications, such as HIV and cystic fibrosis [68,70]. Still, for expanded clinical applications, detailed specificity analyses will be necessary due to the difficulty of accurately predicting TALEN off-target activity [69].

Engineering and optimisation of TALE arrays, linker sequences, and FokI has also produced opportunities for improving specificity and, therefore, the safety of TALENs. Modifying TALE arrays has resulted in an increase in the nucleases’ ability to distinguish between on- and off-target locations [55]. Similarly, modifications in FokI have resulted in improved editing efficiency with reduced off-target activity. For instance, “Sharkey” mutations and ELD obligate heterodimer mutations in FokI have similar properties in TALENs as they do in ZFNs [70], with experiments reporting a 3–6-fold increase in on-target mutation activity. Specifically, the ELD mutation within the FokI nuclease domain results in obligate heterodimerisation being required for cleavage, further increasing on-target specificity [70]. Since off-target activity is partially attributed to the formation of homodimers, obligate heterodimerisation of TALENs can help reduce it [55].

Interestingly, the length of TALE arrays also appears to play a role in specificity. The length is optimal in the range between 17 and 20 bp, with versions below 13 bp associated with toxicity due to nonspecific binding and longer versions potentially increasing the overall tolerance for mismatches and the probability of off-target activity [19,44]. Arrays have also been reported to tolerate up to five mismatches across a standard 19 bp site, with tolerance being higher towards the 3′ end of the sequence [40,41]. Successfully developing TALEN pairs is therefore dependent upon a delicate balance in design: increased array lengths theoretically allow greater specificity but could be more tolerant of mismatches.

Specificity can also be modulated by architecture and structural features. For instance, TALEN architectures that truncate the sequence on both sides of the repeat array (i.e., at both the C- and N-termini) have shown enhanced cleaving activity [40,69] whilst reducing the size of the sequence between monomers, which can assist in limiting off-target activity [45]. Similarly, optimised TALEN scaffolds have shown high DNA cleavage activity only at specific spacer lengths, between 10 and 20 bp in length [40,69]. 

Finally, it is worth considering that off-target activity in TALENs is likely to be related to expression levels. This is due to the high affinity of TALE arrays for their corresponding DNA sequence and the possibility that they may physically saturate on-target sites when expressed at high levels, thus promoting binding of excess monomers to mismatched sequences. This poses another delicate balance: greater expression has been found to increase editing efficiency, but it could also lead to increased off-target effects [70]. 

## 5. CRISPR-Cas

### 5.1. Origin, Structure, and Function

CRISPR-Cas (an acronym for clustered regularly interspaced short palindromic repeats and CRISPR-associated protein) is a powerful genome editing tool originating from bacterial and archaeal adaptive immune responses [71]. Different CRISPR-Cas systems exist, and these vary in the characteristics of the nuclease effector used. This diversity has led to the categorisation of systems into two classes based on structure (single versus multi-subunit) and into six types (I–VI) and 27 subtypes on the basis of specific architecture (modules and functions of each subunit) and loci organisation [72,73]. For instance, class 1 systems consist of types I, III, and IV and feature multi-subunit nuclease effectors. Meanwhile, class 2 systems feature single protein effector modules and consist of types II, V, and VI [74]. The extensively studied, developed and implemented Cas 9 and Cas 12 belong to this latter class, specifically to types II (Cas 9) and V (Cas 12) [72].

Importantly, analysing the use of these systems for versatile genome editing requires understanding their native biological context and mechanism. CRISPR-Cas activity is triggered in response to foreign DNA encounters and involves three distinct stages: adaptation, pre-CRISPR RNA (pre-crRNA) expression/processing, and interference [72,75]. The first stage (performed by Cas1, 2, and 4, depending on the system) involves binding and cleaving a foreign sequence via the introduction of two DSBs after the recognition of a conserved 2–4 bp protospacer adjacent motif (PAM). The resulting cleaved segment (protospacer) is then incorporated into an existent CRISPR array, a collection of partially palindromic repeats and intervening spacers, using cellular repair machinery [72,75].

**Figure 5:**
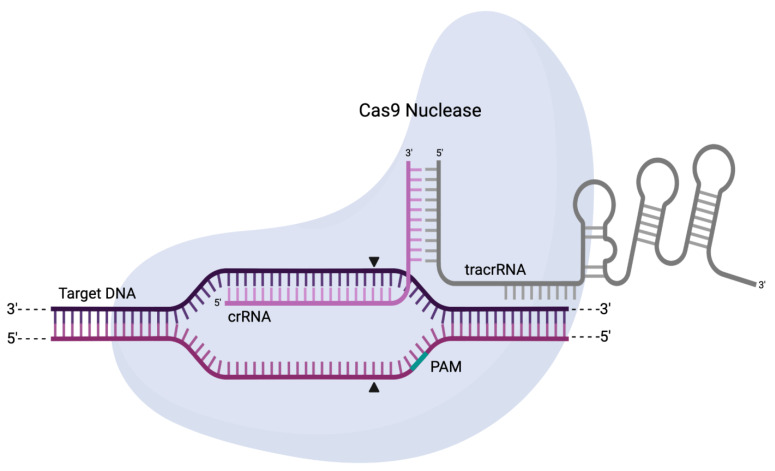
Schematic representation of a Cas9 nuclease in complex with crRNA, tracrRNA, and a target sequence. The crRNA will directly guide the recognition of the target sequence and form a heteroduplex with it while the tracrRNA serves as a structural interface with the nuclease. The highlighted region represents the protospacer adjacent motif (PAM), which is required for sequence recognition and cleavage. The arrows indicate the two separate cuts resulting in a blunt-ended DSB.

Upon further encounters with cognate sequences, the CRISPR array is used as the basis to produce a single transcript called pre-crRNA. Further processing by Cas6, an RNase, or a single large Cas protein with multiple functionalities (e.g., Cas12), results in mature crRNAs that can be used as “guides” during the interference stage. Specifically, in this final stage, the crRNA interacts with transactivating CRISPR RNA (tracrRNA—a structural and interfacing RNA that physically binds to an effector Cas) to lead the entire Cas–tracrRNA complex towards recognisable sequences. Selective binding then allows the inactivation of such sequences through cleavage by the Cas nuclease effector (Figure 5) [72,75,76].

### 5.2. Feasibility

Simplifying the natural process described previously enables its repurposing for versatile genome editing. The focus is entirely on the interference stage, where selective sequence recognition and cleavage occur, and on the components and events involved in it: a Cas nuclease, crRNA, and tracrRNA. In fact, specifically targeting new sequences using CRISPR-Cas is possible by modifying the crRNA that directs a given Cas nuclease, as opposed to reengineering the entire protein [29,77,78]. This distinguishes it from other genome editing platforms such as MNs, ZFNs, and TALENs and has ensured its quick and widespread adoption. The fact that extensive protein engineering (MNs and ZFNs) or complex assembly processes (TALENs) are not necessary to target new sequences makes CRISPR-Cas simultaneously more accessible and versatile [29,79].

Importantly, the central 20-nucleotide crRNA and the accessory RNA sequence that interfaces with the Cas nuclease and provides structural support (tracrRNA) can be combined into a single guide RNA (sgRNA) [55,80,81]. This is common for Cas9, which requires both components, whereas the more recently identified Cas12 only requires a crRNA as its guide [80,82]. Ultimately, the crRNA component is responsible for selective Cas binding by forming a heteroduplex with its complementary sequence in double-stranded DNA (dsDNA). Therefore, altering the sequence of the crRNA changes its complementary DNA sequence, and with it the cleavage site.

Given its crucial role, designing a sgRNA involves a series of important considerations. For instance, the presence of certain bases in specific positions of the sgRNA has been consistently associated with functionality and efficiency in a manner dependent upon the Cas variant [83]. In the case of Cas9, negative selection and depletion assays and comparison of sgRNA performance using the same genes have revealed a strong preference for specific features in the spacer region: purines (particularly guanine) in positions −1, −2, and −4 relative to the PAM (5′–3′ orientation); cytosine in position −3, coinciding with the site of cleavage for Cas9; adenines in positions from −5 to −12; purines (adenine or guanine) in positions from −14 to −19; and guanine in position −20 [83,84,85].

Similarly, adequate design must consider the existent strategies to maximise efficiency and minimise potential off-target activity [86]. Many of these strategies build upon the knowledge gained through years of development and optimisation of small interfering RNAs (siRNAs). As a result, they often involve the use of chemical modifications in specific positions of the sgRNA, such as sugar modifications like 2′-*O*-methyl (2′*O*Me), 2′-*O*-methoxyethyl (2′M*O*E), and 2′-fluoro (2′F), which result in changes in furanose ring conformation that increase binding affinity. Likewise, phosphate backbone modifications such as 3′-phosphorothioate (3′PS) or 3′thiophosphonoacetate linkages (3′thioPACE) enhance nuclease resistance and reduce binding affinity [87]. 

Multiple algorithms and design rule sets have also been established to guide the process of selecting the “optimal” sgRNA for a defined target from a pool of potential candidates. These involve identifying and combining highly favoured sequence features including position-specific nucleotides, global GC count, overall nucleotide counts, target sequence location, as well as predicting off-target activity [88].

However, there are certain constraints inherent to CRISPR-Cas editing. The most prominent of these is the requirement for a PAM, a highly conserved set of bases that must be immediately adjacent to the target sequence, in the 3′ end in Cas9 and the 5′ end in Cas12 [79,89,90]. Crucially, this PAM requirement is not imparted by the sgRNA but is due to affinity contacts between highly conserved residues in the Cas and specific bases in DNA [77,90]. As a result of this, the specific PAM varies as a function of the Cas variant [55,91,92]. Naturally, this currently unavoidable requirement introduces a design constraint when considering new targets: not every sequence will be targetable with a single Cas variant or at all using CRISPR-Cas. This has spurred the search for novel, naturally occurring Cas variants with alternative PAM preferences (e.g., Cas12a, Cas12e, Cas14), as well as re-engineering variants with expanded PAM compatibility or developing possible “PAMless” systems (e.g., SpCas9-NG, xCas9-3.7, and SpRY) [77,90,93]. It is worth noting, however, that while the PAM limits targetable sequences (and thus potential applications), the restriction it imposes may assist in reducing off-target activity.

Beyond the simplicity associated with sgRNA modification, the fact that Cas proteins serve as common effectors also means that multiplexed editing of different sequences is possible by introducing sgRNAs targeting different sequences [55]. Likewise, Cas proteins themselves can be modified to expand the potential applications of the platform. For example, nickase versions (nCas9) have been developed via inactivation of one of the two cleavage active sites of Cas9, allowing the use of the platform for double-nicking strategies [17,80,86]. These involve the use of two nCas9 for the introduction of DSBs with overhangs, with the dual advantages of greater specificity and higher rates of HDR [17,55,86]. 

Catalytically inactive (“dead”) variants (dCas-9 or 12a and 12e) have also become widespread as they allow the highly specific targeting of user-defined sequences without introducing DNA cleavage. This selectivity without intrinsic activity promoted the development of a new set of tools for genome manipulation by enabling fusions with alternative effectors [76,81,94,95]. At its most basic, this is represented by the use of nucleases with distinct cleaving properties (e.g., FokI–dCas9) [80] or even replacement with adenosine and cytidine deaminases capable of driving A-to-G or C-to-T transitions. Crucially, this was at the core of a new direction in CRISPR-Cas genome editing: precise base editing by coupling Cas variants with cytidine and adenosine deaminases [96]. Effectively, the deamination of cytosine and adenosine produces, respectively, uracil and inosine. Under adequate constraints, these are treated as thymine and guanine by replication machinery, thus enabling highly specific A-to-G and C-to-T transitions within short editing windows and without introducing DSBs [97,98]. Importantly, newer versions of both cytidine base editors (CBEs) and adenine base editors (ABEs) use Cas nickase variants (along with a uracil glycosylase inhibitor in CBEs) in order to increase efficiency [96,97,98].

Catalytically inactive Cas variants have also been essential for the development of epigenome editing and transcriptional activation/repression strategies. Case in point, dCas have successfully been fused to a diverse group of histone methyltransferases/acetylases and DNA methyltransferases for the selective modification of epigenetic marks in a target region [79,99,100]. For instance, dCas9 fused to the core domain of acetyltransferase p300 enabled highly precise histone tail acetylation (lysine 27 at histone 3, specifically) at promoter and enhancer sites, resulting in robust gene transcription [101]. Similarly, Tet1 and Dnmt3–dCas9 fusion proteins have been proven effective in selective demethylation and methylation of CpG sites within the promoters of interest [102]. Direct activators and repressors, such as VP64/VPR and Krüppel associated box (KRAB) domains, have also been used in conjunction with dCas9 as part of strategies for transcriptional regulation (CRISPR interference and CRISPR activation) [77,99,100]. 

In addition to these applications, more recently identified Cas variants such as Cas12a and Cas13 have been used as part of highly sensitive multiplexed methods for RNA and DNA detection and quantification with valuable applications in diagnostic screening [79]. Recently, Cas13 has even been used for RNA editing [103,104]. Moreover, the consistently growing list of Cas orthologues is also a significant asset of this genome editing platform. Naturally occurring variants often feature alternative PAM preferences and desirable properties such as smaller size, greater specificity, and biological origins in organisms non-pathogenic to humans, reducing the risk of immunogenic responses [92,105,106].

Ultimately, however, the greatest difficulty for the use of CRISPR-Cas in genome editing may stem from its multiple layers of decision-making. For instance, choosing the most adequate Cas variant for an application or sequence and selecting a specific site to be targeted, both of which can minimise potential off-target activity whilst retaining high editing levels, as well as selecting the best delivery system, the efficiency- and specificity-improving strategies to be implemented, and the specific method of editing to be used (base editing, epigenetic modification, knock-out via indels). However, it is important to note that many of these factors remain true for the other genome editing platforms. Crucially, the increased complexity of decision-making in CRISPR-Cas constitutes not so much an obstacle but an opportunity for rational fine-tuning of performance and a reflection of the significant progress in the field.

### 5.3. Delivery Methods 

The array of delivery options available for all genome editing technologies involves critical decision-making, with potential implications for both safety and efficiency [107,108,109,110]. Moreover, delivery systems pose intrinsic trade-offs in terms of packaging capacity, potential for unintended integration, immunogenicity, and duration of activity. For instance, delivery using plasmid DNA is ideal in terms of cost, lower technical difficulty, and longer expression in cells but results in slower editing as compared to mRNA and protein-based systems besides carrying a high risk of mutagenesis and off-target activity [111]. Meanwhile, adeno-associated viruses (AAVs) are used due to their low immunogenicity and partial integration into the host genome [14], but their low capacity and potential for continued expression hinder their widespread use in clinical applications. Lentiviral vectors (LVs) offer increased packaging capacity but are not compatible with non-recoded TALENs due to their instability [112]. Further challenges with viral delivery systems include gene silencing, improper activity, and misintegration of the transgene [49].

Similar trade-offs are present when choosing the appropriate delivery system for CRISPR-Cas. All components of the system (Cas and sgRNAs) can be introduced into targeted cells through viral and non-viral strategies in the form of DNA, mRNA, or protein complexes, each entailing distinct caveats. Table 5, modelled after the categories identified in [113], provides an overview of the strengths and limitations of each delivery system for CRISPR-Cas. Fundamentally, plasmid-based delivery results in prolonged Cas9 expression in cells, which could increase efficiency, but also the likelihood of off-target effects [114]. In turn, transient Cas expression can be achieved using an mRNA format, but mRNA requires chemical modifications to enhance its stability to avoid fast degradation by RNases [115] that could reduce editing efficiency. Furthermore, near-immediate gene editing can be achieved by the delivery of active Cas nucleases in the protein form. However, the bacterial origins of the relevant variants (like SpCas9 and SaCas9) and the potential immune response they could trigger raises some concerns using this delivery format [107]

### 5.4. Efficiency

Individual modification rates vary significantly as a function of the Cas variant used, sgRNAs implemented, delivery method, improvement strategies, type of editing, and specific sequence targeted. As a result, efficiency analyses are best conducted directly in the context of these parameters. As a point of reference, however, wild-type SpCas9 (a benchmark in the field due to its widespread use and study) has been reported to achieve a mean editing activity of 40–50% on target sites with the canonical NGG PAM as measured by indel frequency detected via high-throughput sequencing (HTS) [77,132]. Some studies even report rates as high as ~73% [90]. It is worth noting that these results were obtained in vitro for HEK293T cells in the context of delivery via plasmid transfection [77,90] and LVs [132]. Other methods of delivery of SpCas9, such as cell-penetrating peptides [133] and ribonucleoproteins (RNPs) [134], resulted in modification frequencies of 16% in HEK293T and 79% in K562 cells, respectively, albeit as measured with T7 endonuclease I (T7E1) assays. 

Crucially, a recent report by Kim et al. (2020) provided one of the most comprehensive comparisons yet of engineered Cas9 variants and orthologues in terms of efficiency and specificity [132]. It compared 13 Cas9 variants across thousands of target sites in HEK293T cells via lentiviral transduction and high-throughput sequencing, thus minimising common sources of variation such as different targets, cell lines, and indel-measuring assays. Overall, they found the average indel frequency for the variants was between 15% and 49% when using the sgRNA that most benefited individual activity (guides with a matched/mismatched guanosine at position −20 or perfectly matched tRNA-N20 sgRNAs). Based on individual results, they were able to rank the “high-fidelity” Cas variants as presented in Table 6. 

Similarly, Kim et al. (2020) were able to evaluate the activities of high-fidelity variants at mismatched target sequences in what can be regarded as an indicator of specificity. Overall, the results highlight a trade-off between activity and specificity. Ultimately, the variants were ranked as presented in Table 7.

### 5.5. Safety 

Both the editing efficiency and the specificity of CRISPR-Cas vary considerably across different genomic sites. Moreover, different orthologues and variants thereof carry different probabilities of base mismatches within both guide RNA and PAM regions, influencing the frequency of off-target activity [135]. As is the case for the other genome editing platforms, sequence similarity of the target to other genomic locations leads to a higher potential for off-target effects. [29]. 

Importantly, CRISPR-Cas can have higher rates of off-target activity when compared to TALENs and ZFNs [29]. This has been linked with a spatially uneven tolerance for pairing mismatches in the sgRNA–DNA heteroduplex: the PAM-proximal region (positions 1–12) is sensitive to mismatches while the distal region (positions 13–20) readily allows single base substitutions in the target sequence [78]. Like ZFNs and TALENs, the activity of the platform is also influenced by the genomic location targeted, with epigenetic modifications, nucleosome occupation of the target, and chromatin context being associated with altered functionality and efficiency [17,86,135,136,137]. For instance, target sites within the open chromatin regions may be mutated more easily by Cas9 than sites with an identical sequence within the closed chromatin regions [86].

The need for precise control mechanisms of the CRISPR-Cas activity constitutes another source of concern when considering highly specific applications, particularly in vivo. This stems from the dual observations that increased off-target effects can arise from prolonged Cas9 activity and that delivery methods that promote shorter, transient presence of Cas proteins and sgRNAs (e.g., RNP complexes) can reduce off-target activity [55,86]. To address this, several promising regulatory mechanisms have been demonstrated (e.g., far-red light-activated split-Cas9 system FAST), and other mechanisms have been proposed based on the recently described anti-CRISPR proteins identified in phages [17,74,138,139]. This has allowed researchers to design and propose “stimulus-, tissue-, organ-, developmental stage-specific” inactivation mechanisms for greater control of genome editing [74]. Additional strategies known to minimise off-target effects, specifically in the CRISPR-Cas9 system, are discussed in Table 8.

Crucially, CRISPR-Cas has recently raised several questions around safety beyond its off-target activity rates. The consistent findings of pre-existing adaptive immune responses in the human population to Cas orthologues derived from common human pathogens (SaCas9 from *Staphylococcus aureus* and SpCas9 from *Streptococcus pyogenes*) highlight a challenge for clinical applications. For instance, Charlesworth et al. (2019) detected antibodies against SaCas9 and SpCas9 in a significant proportion of donors from a sample of healthy adults (67% and 42%, respectively). Moreover, an elevated prevalence of antigen-specific T cells against SaCas9 and SpCas9 was also documented in the donors (78% and 67%, respectively) [71]. These findings underlie the concern that the cells modified using the technology and which continue to express either orthologue could be actively targeted by a patient’s immune system, rendering the treatment inefficient at best and potentially inducing significant toxicity at worst [71]. They also point to the potential for other unexpected immune responses arising from the CRISPR-Cas use due to high frequency of human contact with microorganisms. With public genome repositories from which novel CRISPR-Cas systems are drawn containing a biased skew of pathogenic bacteria and model organisms [152], immunogenicity ought to be a major safety consideration.

Finally, the finding that Cas-induced DSBs trigger a p53-mediated response with high toxicity rates in human pluripotent stem cells (hPSCs) [153] highlights a significant concern for ex vivo therapies using CRISPR-Cas. Since functional p53 reduces editing efficiency in hPSCs and plays a prominent role in tumour suppression, it is worth considering whether successfully modified cells might have inadvertently acquired mutations in p53 [153]. If such is the case, modified cells may be more tolerant of further DNA damages, with corresponding implications for cancer. Therefore, as discussed by Ihry et al. (2018), “it will be critical to ensure that patient cells have a functional p53 before and after engineering” [153].

## 6. Conclusions

### 6.1. Focus and Expectations Are on CRISPR-Cas, but Do Not Discount the Other Platforms

Currently, no single platform is “ideal” or even applicable to every case as it is the context that dictates the best approach for genetic modification within a target organism. Each has significant constraints, but also strengths that could make them suitable for an application. For instance, the high specificity that characterises meganucleases can be adequate for circumstances requiring high precision and where efficiency is not critical. Similarly, ZFNs or TALENs whose FokI domain bears mutations attenuating their cleavage kinetics can produce highly efficient and specific modification of target sequences.

However, all of these technologies are constrained by a cumbersome retargeting process that makes them impractical as a platform technology upon which to build a portfolio of genome editing applications. That is, developing a single variant with any of these technologies for a specific application would be feasible and perhaps advisable, but it may not be strategic when the objective is to continually target new sequences. Furthermore, there are clear differences in versatility and future potential among the technologies assessed. MNs, ZFNs, and TALENs have uneven “degrees of support” noticeable in terms of research, groups, characterisation of parameters, bioinformatic tools, and data from clinical applications.

For its part, CRISPR-Cas features high versatility, with retargeting possible simply by modifying the sgRNA. The diversity of Cas variants with specific parameters and even functionality (e.g., base editors, transcriptional activators/repressors) also provides significant depth to the platform. Moreover, it has been proven compatible with most delivery vehicles, from plasmids to viral vectors and RNPs. 

### 6.2. If CRISPR-Cas Is to Be Used, Consider High-Fidelity Variants of SpCas9 

In the context of CRISPR-Cas, ensuring safety in highly sensitive applications requires choosing variants with the lowest off-target activity rates. While this is often accompanied by lower modification rates, lower efficiency can be addressed in ex vivo applications through selection strategies.

SpCas9 has so far been explored most extensively, revealing its mechanism of action, interactions, strengths, and weaknesses in detail. The recently reported prevalence of adaptive immunity to SpCas9 in human populations poses a potentially high degree of risk due to the origin of the protein in human pathogenic microorganisms. Nevertheless, highly specific variants such as EvoCas9 and SpCas9-HF1 should be regarded as standards of specificity to aspire to with novel variants from non-pathogenic organisms. EvoCas9 features the highest specificity among the high-fidelity SpCas9 variants, and the mutations that produce SpCas9-HF1 are versatile enough to be compatible with PAM-relaxed variants.

### 6.3. Despite Its Versatility, CRISPR-Cas Also Faces Limitations of Its Own

Whilst adapting the platform to new sequences is made simple by sgRNA redesigning, the PAM requirement inevitably limits the number and location of targetable sequences across the genome. This issue is particularly unfavourable to applications requiring highly specific activity and careful positioning of the sgRNA. Such is the case of base editing where base transitions occur in a narrow window near the PAM. 

While the diversity of naturally occurring Cas nucleases and the stride towards PAMless versions can assist in addressing this constraint, all the existing Cas variants possess trade-offs, and the selection process for any new application should reflect this: (1) size influences the packaging method and the delivery system; (2) higher specificity may be accompanied by reduced applicability across diverse sequences; (3) reduced PAM restrictions can result in increased off-target activity. Therefore, currently, no Cas variant will be suitable for every application, and not every sequence may be targeted with a single Cas variant or at all using CRISPR-Cas.

### 6.4. When Deciding on Which Genome Editing Platform to Use, Assess All the Features Related to Safety, Not Only Off-Target Activity

Comprehensively assessing off-target activity requires moving beyond the heterogenous, non-standardised approach prevalent in published research to a standard pipeline, from computational prediction to unbiased detection methods and finally to targeted methodologies. As is the case with Cas variants, the best off-target detection methodology is context-dependent, and the best approach is to generate a comprehensive strategy with multiple methods for cross-validation.

However, the prevalence of adaptive immunity to SpCas9 should lead to including the biological origin in safety considerations for all species and to closer monitoring of other “hidden” risks. Such is the case of potentially “defective” DNA repair pathways (e.g., p53), which may be inadvertently selected in the context of genome editing. Making sure these pathways function in the modified cells before and after editing should be a priority.

### 6.5. Independently Validate Parameters of the Selected Platform Prior to Commitment

The lack of standardisation across published research hinders the field by preventing a fair comparison between platforms and between specific variants. Different cell lines, delivery systems, genes, and target sequences within those genes all add noise to intrinsic differences. Often, this results in contradictions between different groups examining the same platform (or, in the case of CRISPR-Cas, the same variant) and is compounded by a lack of wide-ranging experimental comparisons. As a result, we consider establishing a standardised process for “in-house” validation of specificity and efficiency should be a priority before committing logistical, legal, commercial, or R&D resources to a platform.

## Figures and Tables

**Table 1:** Modification rates obtained using MNs in clinically relevant applications.

Application	Modification Rate/Gene of Interest	Delivery Ssystem and Modification Target	Meganuclease
Recessive dystrophic epidermolysis bullosa (RDEB)	9% modification (indel formation) of *COL7A1* in RDEB-K-SV40 cells	Integrase-deficient lentiviral vector (IDLV)	MN-i.1 lentiviral (I-CreI-derived MN isoschizomer targeting intron 2 of *COL7A*1) [21]
	7.5% modification (indel formation) of *COL7A1* in RDEB-K (primary keratinocytes)		
	2.2% modification (indel formation) of the *COL7A1* gene in RDEB-F (primary fibroblasts)		
Severe combined immunodeficiency (SCID)	Gene correction events of *RAG1* in 5.3% of transfected cells	Plasmid in human 293H cells	RAG1 MN (single-chain I-CreI variant) [25]
	Gene insertion for repairing *RAG1* in up to 6% of transfected cells		RAG1 MN (single-chain I-CreI variant) [23]
Xeroderma pigmentosum group C (XPC)	High specificity in cleaving the *XPC* locus without apparent genotoxicity or evidence of off-target activity (specific rates not presented as percentages)	Lipofection in CHO-p10_XPC2 cells (efficiency) and human MRC5 cells (specificity)	Engineered variants of I-CreI (Ini3-Ini4 and Amel3-Amel4) [26]
Duchenne muscular dystrophy (DMD)	13% and 30% expression of the corrected *DMD* gene (as compared to a positive control) using I-Scel and RAG1, respectively	Lipofection in 293FT cells	I-Scel and RAG1 [27]
Prevention of graft-versus-host disease	1.6% disruption (indel formation) of the *TCRα* gene (with TCRα MN) 70.4% disruption (indel formation) of the *TCRα* gene (with TCRα MegaTAL)	Messenger RNA (mRNA) encoding the indicated constructs in human primary T cells	TCRα MN (I-OnuI variant engineered to knock-out *TCRα*) and TCRα megaTAL [19]

**Table 2:** Strategies for the assembly and selection of new ZFNs.

Strategy	Description	Strengths and Weaknesses
Modular assembly [32]	Phage display-based. Seeks to identify individual ZFs with an established affinity for certain base triplets from an existing archive and link them together.	Reduces sequence specificity, binding affinity, and efficacy. Higher toxicity.
Oligomerised pool engineering (OPEN) [32]	Pre-established ZFNs, randomly assembled via PCR from a pool of ZFs, are screened against the target, and selected in a bacterial two-hybrid system.	Produces one of the highest specificities but requires significant time, labour and expertise.
Context-dependent assembly (CoDA) [32]	Targets a new sequence by exchanging ZFs between the already validated ZFNs that share a common middle ZF. Adequate for 3-ZF nucleases.	ZFNs produced with CoDA are less specific than those produced with OPEN, but the process is less technically demanding.
2 + 2 [32]	4-ZF nucleases are built by combining discrete 2-ZF subunits with known affinities, followed by optimisation.	Developed by Sangamo Biosciences and available commercially.
Sequential context-sensitive selection [42]	Uses transcription factor Zif268 as the starting framework and phage display for selection. Each ZF motif undergoes randomisation of six base-contacting residues and is progressively incorporated and optimised for target sequence and context before moving on to the next motif.	An early method for the retargeting of ZFNs. Due to its multiple selection rounds and emphasis on stepwise optimisation, it may be labour- and expertise-intensive. Outdated as compared to OPEN and CoDA.
Bipartite library [43]	Phage display-based. It uses two complementary libraries, each encoding a 3-ZF domain based on the transcription factor Zif268. One library features randomisations in base-contacting residues for ZF motifs 1 and 2, and the other for ZF motif 3.	Early strategy for the development of ZFNs. Outdated with regards to the more prevalent OPEN and CoDA strategies.

**Table 3:** Modification rates obtained using ZFNs in clinically relevant applications.

Application	Modification Rate/Gene of Interest	Delivery System	Modification Target
Human immunodeficiency virus (HIV)	Disruption of *CCR5* with a frequency of 17%	Electroporation	CD34+ hematopoietic stem and progenitor cells (HSPC) [51]
HIV-1 resistance	>50% disruption frequency of *CCR5*	Adenoviral vector	GHOST-CCR5 cell line [52]
X-linked SCID	6.6% homozygous cells with a modified *IL2Rγ* locus	Transfection and electroporation	K562 cell line [49]
X-linked SCID	29% disruption frequency of *IL2Rγ*	IDLV	K562 cell line [31]
Sickle cell anemia	37.9% modification rate of the *β-globin* gene	Electroporation	Human induced pluripotent stem cells (iPSCs) [53]
Leber congenital amaurosis	85% indel frequency in the *CEP290* gene	Messenger RNA (mRNA) delivery	K562 cells [46]

**Table 4:** Efficiency rates and modifications obtained using TALENs.

Application	Modification/Gene of Interest	Delivery System	Modification Target
HIV-1 infection (*CCR5*)	50.4% targeted mutation frequency of *CCR5* without selection; homologous recombination in 8.8% of the targeted cells (to *CCR5Δ32*).	Electroporation	CD4 + U87 cells [62]
Sickle cell disease (SCD)	Correction of mutation E6V in the *HBB* gene via HDR and a donor sequence; >60% of hiPSC colonies correctly targeted.	Electroporation	Patient-derived human induced pluripotent stem cells (hiPSCs) [63]
Alpha-1 antitrypsin (AAT) deficiency	Correction of AAT Z mutation via HDR and a donor sequence; 25–33% biallelic targeting efficiency.	Electroporation	Patient-derived iPSCs with AAT deficiency [64]
Recessive dystrophic epidermolysis bullosa (RDEB)	Gene correction of *COL7A1* via HDR and a donor sequence. Enables normal protein expression in a teratoma-based skin model in vivo.	Electroporation	Primary fibroblasts that were reprogrammed into iPSCs [65]
Comparison of specificity and cytotoxicity across human loci (*CCR5*, *AAVS1*, and *IL2RG*)	6–17% allelic mutation frequency: *CCR5* (7%), *AAVS1* (6%), *IL2RG* (17%).	Electroporation	Primary human newborn foreskin fibroblasts (NuFFs) [41]
Editing of oncoprotein E7 from human papillomavirus (HPV)	~10% editing efficiency of E7 accompanied by complete silencing.	Lipofection	SiHa cells [66]
Safe harbour-mediated knock-in in bovine cells	70% knock-in efficiency (*bRosa26* locus).	Electroporation	Bovine fetal fibroblasts (BFFs) [67]

**Table 5:** Summary of the most common delivery systems in CRISPR-Cas systems.

	Strategy	Form of Delivery	Strengths	Limitations
Viral delivery	Adeno-associated viral vectors (AAV)	DNA	No genome integration, low immunogenicity and high potential for in vivo applications with transient gene expression [116,117].	Low capacity for cloning (<4.7 kb). The common strain of Cas9 from *Streptococcus pyrogenes* is a less feasible option due to its large size (~4.2 kb). Its efficiency in gene targeting is still low.
	Lentiviral vectors (LV)	DNA	Higher capacity than AAV (<8 kb) with high efficiency across different cell types [108].	Tumorigenesis concerns due to the activation of oncogenes by the random integration into the genome of the host cell [117,118].
	Adenovirus (AV)	DNA	High transduction efficiency and broad tropism. No integration into host cells. Extensively studied for clinical trials [117].	Laborious process for the production of AVs [119]. Pre-existing immunity to multiple AV serotypes [117]. Causes inflammation of tissues due to the innate immune response by its delivery [120].
	Extracellular vesicles (EV)	Protein	No integration into the host genome as EVs do not contain any viral genome. Higher safety due to transient activity resulting in low off-target effects [113]. Intrinsic durability, tolerability, and potential for cell type-specific targeting [121].	Quantification methods are limited. Significant need for standardisation of isolation and analytical procedures [121]. Protease cleavage in Cas9 may occur, which leads to its degradation [122].
Non-viral delivery	Microinjection	DNA, mRNA, or protein	Direct delivery into cells under controllable parameters. No capacity limitations for Cas9 delivery into the nucleus.	Laborious, low-throughput, requires a microscope for injection, and is not compatible with in vivo applications [123].
	Electroporation		Well-established methodology that has been proven efficient across a variety of cell types [110].	Specialised equipment and potentially costly. Cell viability can be affected by the high electrical current. Not suitable for a variety of cell types due to sensitivity to stress.
	Cell-penetrating peptide (CPP)	Protein	No random integration into the host genome. Its versatility enables a variety of cargoes to be delivered as complexes into cells [124].	Variable efficiency requiring extensive optimisation [125]. Low stability and potential immunogenicity in vivo coupled with low intrinsic specificity [126].
	Lipid-based nanoparticles (LNPs)	DNA, mRNA or Protein	High versatility, large capacity, minimised concerns of immunogenicity, extensive testing across clinical trials [127].	Significant tailoring and optimisation of composition to maintain minimal toxicity and high efficiency for different routes of administration and cell types [127,128]. Low efficiency compared to viral delivery and electroporation [129].
	Gold nanoparticles	Protein	Multiple controllable parameters, from size to surface functionalisation [130]. Nonimmunogenic responses with higher efficiency compared to LNPs [109].	Potential for toxicity from residual contaminants (derived from conventional production) or stabilising agents [131]. Further research is required.

**Table 6:** Ranking of high-fidelity Cas9 variants according to efficiency. Adapted from Kim et al. (2020) [132].

Rank	Cas Variant	Average Indel Frequency	Comparison with the Pprevious Rank
1	SpCas9	49%	-
2	Sniper-Cas9	46%	≤
3	eSpCas9(1.1)	40%	<
4	SpCas9-HF1	34%	<
5	xCas9	32%	≤
6	HypaCas9	30%	≈
7	EvoCas9	15%	<<

**Table 7:** Ranking of high-fidelity Cas9 variants according to specificity. Adapted from Kim et al. (2020) [132].

Rank	Cas Variant	Specificity 1–(Indel Frequencies at the Mismatched Target Sequences Divided by Those at the Perfectly Matched Targets)	Comparison with the Previous Rank
1	EvoCas9	0.89	-
2	HypaCas9	0.67	<<
3	SpCas9-HF1	0.58	≤
4	eSpCas9(1.1)	0.50	≈
5	xCas9	0.42	<
6	Sniper-Cas9	0.36	<
7	SpCas9	0.35	<

**Table 8:** Mitigation strategies and potential improvements for off-target activity in CRISPR-Cas (adapted from [86], which offers an excellent review on these strategies).

Mitigation Strategy	Description	Improvement
Truncated guide RNAs (gRNAs)	17–18 (instead of 20) nucleotides complementary to the target site	Reduced off-target indels (up to 5000-fold) without sacrificing the efficiency of desired edits [140]
Chemical modification of gRNA	Incorporation of bridged nucleic acids into crRNA	Reduced off-target cleavage (up to 24,000-fold (site-dependent)) [141]
RNP delivery	RNA-guided engineered nuclease and gRNA are complexed for a direct delivery into cells	Compared to the plasmid delivery, reduced off-target indels (around 10-fold) and unwanted chromosomal rearrangements without sacrificing editing efficiency due to the rapid degradation (within 24 h) of the RNP in cells [134]
RNA-guided FokI–dCas9 nucleases (RFNs)	Fusion of dCas9 to the FokI nuclease (fCas9); requires functional dimers to cleave target DNA	On-target-to-off-target ratio (specificity) 140-fold higher than that of WT Cas9 [142] Further increase with truncated gRNA [143]
Paired Cas9 nickases	Double nicking with D10 (nuclease domain)	Production of indels at known off-target sites below the detection limit of 0.1%; increased differentiation of highly similar off-target sites (160- to 990-fold increase in on-target-to-off-target activity) [144]; minimises detectable off-target sites as assessed via HTGTS [145]
Split SpCas9	Separates the two structural lobes comprising Cas9 (α-helical and nuclease) into distinct polypeptides to control assembly and activity [146]; Cas9 can also be split at suitable sites with the resulting fragments bound to rapamycin-binding domains (FRP, FKBP) to enable inducible dimerisation [147]	Lowers cleaving efficiency but promotes higher specificity [146]
Programmable DNA-binding domain–Cas chimera (Cas9–pDBD)	Programmable DNA-binding domain system—fusion of the ZF protein to SpCas9 increases the recognition site length	Up to 150-fold increase in the specificity ratio (on-target-to-off-target activity) [148]
Structure-based design	1-eSpCas9(1.1) (enhanced *Streptococcus pyrogenes* Cas9): structure-guided design weakens the binding affinity to the nontarget DNA strand; this improves specificity by reducing binding stability at off-target sites whilst maintaining on-target activity [149] 2-SpCas-HF1 (high fidelity): reduced cleaving ability at off-target sites enabled by disrupting residues that form hydrogen bonds with the DNA backbone (thus limiting stability at mismatched sequences) [150]	
	3-EvoCas9: a Cas9 variant with four beneficial mutations resulting in a 79-fold specificity improvement compared to wild-type SpCas9 [151] 4-xCas9: broadened PAM recognition that supports an expanded sequence targeting capability with minimal off-target activity [77]	
Anti-CRISPR	Ability to prevent the expression of Cas proteins, block cleavage activity and the CRISPR-Cas complex assembly, and inhibition of crRNA transcription and processing [74]	

## Data Availability

Not applicable.

## Abbreviation

TALEN	Transcription activator-like effector nuclease
CRISPR-Cas	Clustered regularly interspaced short palindromic repeats-Cas
DSB	Double-strand break
NHEJ	Nonhomologous end joining
HDR	Homology-directed repair
Indel	Nucleotide insertion and deletion
MN	Meganuclease
HE	Homing endonuclease
bp	Base pair
IVC	In vitro compartmentalised system
MT	MegaTAL
TALE	Transcription activator-like effector
RDEB	Recessive dystrophic epidermolysis bullosa
TCRα	T cell receptor alpha
RDEB-K	Primary recessive dystrophic epidermolysis bullosa keratinocyte
RDEB-F	Primary recessive dystrophic epidermolysis bullosa fibroblast
IDLV	Integrase-deficient lentiviral vector
SCID	Severe combined immunodeficiency
XPC	Xeroderma pigmentosum group C
DMD	Duchenne muscular dystrophy
ZFN	Zinc finger nuclease
ZF	Zinc finger
C2H2	Cys2/His2
OPEN	Oligomerised pool engineering
CoDA	Context-dependent assembly
AAV	Adeno-associated virus
LV	Lentiviral vector
HIV	Human immunodeficiency virus
HSPC	Hematopoietic stem and progenitor cell
iPSC	Induced pluripotent stem cell
RVD	Repeat variable diresidue
RVR	Repeat variable residue
SCD	Sickle cell disease
hiPSC	Human induced pluripotent stem cells
AAT	Alpha-1 antitrypsin deficiency
AAVS1	Adeno-associated virus integration site 1
NuFF	Newborn foreskin fibroblast
HPV	Human papillomavirus
bRosa26-EGFP	Bovine rosa26-enhanced green fluorescent protein
RMCE	Recombinase-mediated cassette exchange
HTGTS	High-throughput genome-wide translocation sequencing
Cas	CRISPR-associated protein
crRNA	CRISPR RNA
PAM	Protospacer adjacent motif
tracrRNA	Trans-activating CRISPR RNA
sgRNA	Single guide RNA
dsDNA	Double-stranded DNA
siRNA	Small interfering RNA
2′OMe	2′-O-methyl
2′MOE	2′-O-methoxyethyl
2′F	2′-fluoro
3′PS	3′-phosphorothioate
3′thioPACE	3′thiophosphonoacetate linkage
nCas9	Cas9 nickase
dCas9	Dead Cas9
HTS	High-throughput sequencing
RNP	Ribonucleoprotein
T7E1	T7 endonuclease I
FAST	Far-red light-activated split-Cas9 system
gRNA	Guide RNA
RFN	RNA-guided FokI–dCas9 nuclease
Cas9-pDBD	Programmable DNA-binding domain
hPSC	Human pluripotent stem cell
